# *TNFRSF1B *A1466G genotype is predictive of clinical efficacy after treatment with a definitive 5-fluorouracil/cisplatin-based chemoradiotherapy in Japanese patients with esophageal squamous cell carcinoma

**DOI:** 10.1186/1756-9966-29-100

**Published:** 2010-07-20

**Authors:** Akiko Kuwahara, Motohiro Yamamori, Megumi Fujita, Tatsuya Okuno, Takao Tamura, Kaori Kadoyama, Noboru Okamura, Tsutomu Nakamura, Toshiyuki Sakaeda

**Affiliations:** 1 School of Pharmacy and Pharmaceutical Sciences, Mukogawa Women's University, Nishinomiya 663-8179, Japan; 2 Graduate School of Pharmaceutical Sciences, Kyoto University, Kyoto 606-8501, Japan; 3 Kobe University Graduate School of Medicine, Kobe 650-0017, Japan

## Abstract

**Background:**

Currently definitive 5-fluorouracil (5-FU)/cisplatin (CDDP) -based chemotherapy is recognized as one of the most promising treatments for esophageal cancer. A series of studies performed found genetic polymorphisms and the plasma concentration of 5-FU to be predictive of acute severe toxicities and clinical response. Genetic polymorphisms of *tumor necrosis factor (TNF) -α *and its surface receptors, *TNFRSF1A *and *TNFRSF1B *have been examined in terms of susceptibility to various cancers. In this study, genetic polymorphisms of *TNFRSF1B *gene were evaluated Japanese esophageal squamous cell carcinoma (ESCC) patients treated with the definitive 5-FU/CDDP-based chemoradiotherapy and their predictive values of prognosis or severe acute toxicities were assessed.

**Methods:**

Forty-six patients with ESCC were treated with the definitive 5-FU/CDDP-based chemoradiotherapy, one course of which consisted of the continuous infusion of 5-FU for days 1-5 and 8-12, the infusion of CDDP on days 1 and 8, and the radiation at 2 Gy/day on days 1-5, 8-12, and 15-19, with a second course repeated after 2-week interval. Genetic polymorphisms of a TNF-α receptor *TNFRSF1B *gene were determined by a TaqMan^® ^MGB probe-based polymerase chain reaction.

**Results:**

The genotype of *TNFSR1B *A1466G, but not M196R/T587G or C1493T, was found to be predictive of clinical response, i.e., a complete response or not (p = 0.040). Clinical response was predicted by tumor size (p = 0,002), lymph node metastasis (p = 0.007), distant metastasis (p = 0.001) and disease stage (p < 0.001), but *TNFRSF1B *A1466G genotype was independent of these factors.

**Conclusions:**

Genetic polymorphism of *TNFRSF1B *A1466G was found to be predictive response in Japanese ESCC patients with a definitive 5-FU/CDDP-based chemoradiotherapy. Further clinical investigation with a large number of patients or experiments in vitro should be performed to assess the predictive value of *TNFRSF1B *A1466G genotype after chemoradiotherapy.

## Background

A clinical report published in 1999, the RTOG (Radiation Therapy Oncology Group) 85-01 trial involving 134 patients with T1-3, N0-1 and M0 esophageal cancer, is of great interest in terms of clinical outcome because it demonstrated a 5-year survival rate of 26% [1]. This treatment consists of infusions of 5-fluorouracil (5-FU) and cisplatin (CDDP), and concurrent radiation, without pre- or post-surgical resection. Simultaneously in Japan, a modified version was proposed by Ohtsu and his co-workers for advanced metastatic esophageal cancer [2,3]. Two independent clinical investigations have shown curative potential using this regimen for unresectable esophageal squamous cell carcinoma (ESCC) of T4 or M1a [2,3]. A long-term evaluation of efficacy and toxicity with 139 patients revealed a complete response (CR) rate of 56%, along with a 5-year survival rate of 29% [4,5]. Currently, definitive 5-FU/CDDP-based chemoradiotherapy is recognized as one of the most promising treatments for esophageal cancer [6]. A series of studies performed to find a marker predictive of clinical outcome after treatment with a definitive 5-FU/CDDP-based chemoradiotherapy found a genetic polymorphism, G-1154A, of vascular endothelial growth factor to be a predictor of severe acute leukopenia and cheilitis, and the plasma concentration of 5-FU to be predictive of clinical response [7-9].

Tumor necrosis factor (TNF)-α, a proinflammatory cytokine, plays a key role in the pathogenesis of inflammatory diseases. Its biological effects are elicited by binding to its two cognate cell surface receptors, TNFRSF1A/TNFR1 (p55/60) and TNFRSF1B/TNFR2 (p75/80), both of which are involved in increasing expression of other cytokines and immuno-regulatory molecules through the activation of nuclear factor κB. Through extensive examinations of expression and function, some genetic variations have been shown to explain inter-individual variation. Single nucleotide polymorphisms (SNPs) in the *TNF-α*, *TNFRSF1A *and *TNFRSF1B *genes have been identified, however functional data pertaining to these polymorphisms in scarce. Nonetheless, the putative role of these polymorphisms in disease susceptibility has been examined in genetic association studies of various inflammatory disorders, including Crohn's disease [10-13], ulcerative colitis [10,11,14], systemic lupus erythematosus [15-17] and rheumatoid arthritis [18,19]. More recently, given that cancer progression is preceded by a long period of subclinical inflammation [20-22], the genetic polymorphisms of *TNF-α*, *TNFRSF1A *and *TNFRSF1B *have been examined in terms of susceptibility to various cancers [23-28]. In this study, genetic polymorphisms of the *TNFRSF1B *gene, M196R/T587G, A1466G and C1493T, were evaluated in Japanese ESCC patients treated with a definitive 5-FU/CDDP-based chemoradiotherapy, and their predictive values of prognosis or severe acute toxicities were assessed. To our knowledge, this is the first paper to report that the *TNFRSF1B *genotype is predictive of the clinical efficacy of cancer chemoradiotherapy.

## Methods

### Patients

Forty-six male ESCC patients were enrolled in this study based on the following criteria: 1) ESCC treated with a definitive 5-FU/CDDP-based chemoradiotherapy at Kobe University Hospital, Japan, from August 2002 to June 2006; 2) clinical stage T1 to T4, N0 or N1, and M0 or M1a according to the International Union Against Cancer tumor-node-metastasis (TNM) classification; 3) age less than 85 years; 4) an Eastern Cooperative Oncology Group performance status of 0 to 2; 5) adequate bone marrow, renal, and hepatic function; 6) no prior chemotherapy; 7) no severe medical complications; and 8) no other active malignancies (except early cancer). The tumors were histologically confirmed to be primary, and no patients with recurrence were included in this study. Written informed consent was obtained from all participants prior to enrollment. This study was conducted with the authorization of the institutional review board and followed the medical research council guidelines of Kobe University.

### Protocol

**Figure 1:**
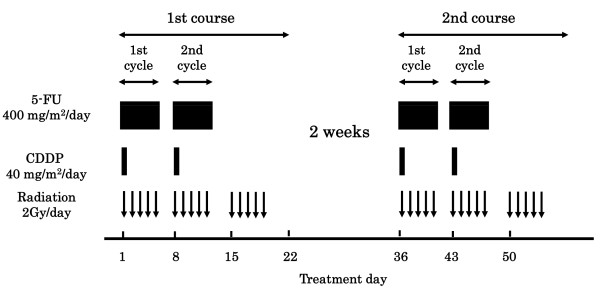
**Protocol of a definitive 5-fluorouracil/cisplatin-based chemoradiotherapy**. One course of treatment consisted of protracted venous infusions of 5-FU (400 mg/m^2^/day, days 1-5 and 8-12) and CDDP (40 mg/m^2^/day, days 1 and 8), and radiation (2 Gy/day, days 1-5, 8-12, and 15-19), with a second course (days 36-56) repeated after a 2-week interval.

The protocol is presented in Figure 1. A course consisted of the continuous infusion of 5-FU at 400 mg/m^2^/day for days 1-5 and 8-12, the infusion of CDDP at 40 mg/m2/day on days 1 and 8, and the radiation at 2 Gy/day on days 1 to 5, 8 to 12, and 15 to 19, with a second course repeated after a 2-week interval [2,3]. If disease progression/recurrence was observed, either salvage surgery, endoscopic treatment, or another regimen of chemotherapy was scheduled.

### Genotyping

Genomic DNA was isolated from whole blood with a TaqMan^® ^Sample-to-SNP™ kit (Applied Biosystems, Foster City, CA, USA) according to the manufacturer's directions. Genetic polymorphisms of *TNFRSF1B*; M196R/T587G, A1466G and C1493T, were determined by a TaqMan^® ^MGB probe-based polymerase chain reaction (PCR) using the StepOne™ real-time PCR system (Applied Biosystems) and pre-manufactured TaqMan^® ^SNP genotyping assays C_8861232_20 (M196R/T587G, rs1061622), C_8861229_10 (A1466G, rs1061624) and C_8861228_20 (C1493T, rs3397) (Applied Biosystems). The PCR was carried out according to the manufacturer's protocol. For each set of reactions, DNA of cases and controls was taken and a negative control containing H_2_O instead of DNA was added to check for contamination.

### Clinical response

The clinical response was evaluated according to the method reported previously [2-5]. Briefly, a CR was defined as the complete disappearance of all measurable and assessable disease at the first evaluation, which was performed 1 month after the completion of chemoradiotherapy to determine whether the disease had progressed. The clinical response was evaluated by endoscopy and chest and abdominal computed tomography (CT) scans in each course. A CR at the primary site was evaluated by endoscopic examination when all of the following criteria were satisfied on observation of the entire esophagus: 1) disappearance of the tumor lesion; 2) disappearance of ulceration (slough); and 3) absence of cancer cells in biopsy specimens. If small nodes of 1 cm or less were detected on CT scans, the recovery was defined as an "uncertain CR" after confirmation of no progression for at least 3 months. An "uncertain CR" was included as a CR when calculating the CR rate. When these criteria were not satisfied, a non-CR was assigned. The existence of erosion, a granular protruded lesion, an ulcer scar, and 1.2 w/v% iodine/glycerin-voiding lesions did not prevent an evaluation of CR. The evaluations were performed every month for the first 3 months, and when the criteria for CR were not satisfied at 3 months, the result was changed to non-CR. Follow-up evaluations were performed thereafter every 3 months for 3 years by endoscopy and CT scan. After 3 years, patients were seen every 6 months. During the follow-up period, a routine course of physical examinations and clinical laboratory tests was performed to check the patient's health.

### Severe acute toxicities

Definitive 5-FU/CDDP-based chemoradiotherapy is associated with acute toxicities; leucopenia, anemia, thrombocytopenia, nausea/vomiting, diarrhea, mucositis (including stomatitis), esophagitis, and renal dysfunction [2-5]. Here, severe acute leucopenia, stomatitis, and cheilitis were subjected to analysis. Toxicity was evaluated using criteria defined by the Japan Clinical Oncology Group [29]. These criteria were based on the National Cancer Institute Common Toxicity Criteria. Toxicity was assessed on a 2 to 3-day basis during the chemoradiotherapy and subsequent hospitalization period and on every visit after the completion of chemoradiotherapy. Episodes of leucopenia, stomatitis, and cheilitis during the first 2 courses and subsequent 2 weeks (until day 70) were recorded as acute toxicities and those of grade 3 or more as severe acute toxicities.

### Survival after the chemoradiotherapy

The survival period was defined as the time from the date of treatment initiation to that of death from any causes or to the last date of confirmation of survival. Survival data were updated on December 31, 2006, and the 2-year survival rate was assessed using the data for 36 patients.

### Data analysis and statistics

All values reported are the mean ± standard deviation (SD). The unpaired Student's *t*-test/Welch's test or Mann-Whitney's *U *test was used for two-group comparisons of the concentrations. Fisher's exact test was used for the analysis of contingency tables. The difference of overall survival curves was analyzed by Log-rank test. P values of less than 0.05 (two tailed) were considered to be significant.

## Results

Demographic and clinicopathologic characteristics of the 46 ESCC patients are summarized in Table 1. The ratio of T1/T2/T3/T4 was 15/6/14/12, that of N0/N1 was 21/25, and that of M0/M1a was 39/7, resulting in a stage I/II/III/IVa ratio of 12/10/17/7. The CR rate was 47.8% (22/46), and 2-year survival rate was 50.0% (18/36). The clinical response, i.e., CR or non-CR, was predicted by T class (p = 0.002), N class (p = 0.007), M class (p = 0.001) and disease stage (p < 0.001). Episodes of severe acute leucopenia, stomatitis and cheilitis occurred in 39.1% (18/46), 13.0% (6/46) and 15.2% (7/46) of cases, respectively and no associations were found with the demographic and clinicopathologic characteristics.

**Table 1:** Demographic and clinicopathologic characteristics of Japanese patients with esophageal squamous cell carcinoma.

Age, yr	64.6 ± 7.2 (range 48-78)
Height, cm	164.2 ± 6.2 (range 152-180)
Weight, kg	56.7 ± 9.6 (33-79)
Male/Female	46/0
Performance status, 0/1/2/unknown	23/19/3/1
Differentiation, well/moderate/poor/unknown	7/27/6/6
T1/T2/T3/T4	15/6/14/12
N0/N1	21/25
M0/M1a	39/7
Stage I/II/III/IVa	12/10/17/7

The values are the mean ± SD. Noncervical primary tumours with positive supraclavicular lymphnodes were defined as M1a.

**Figure 2:**
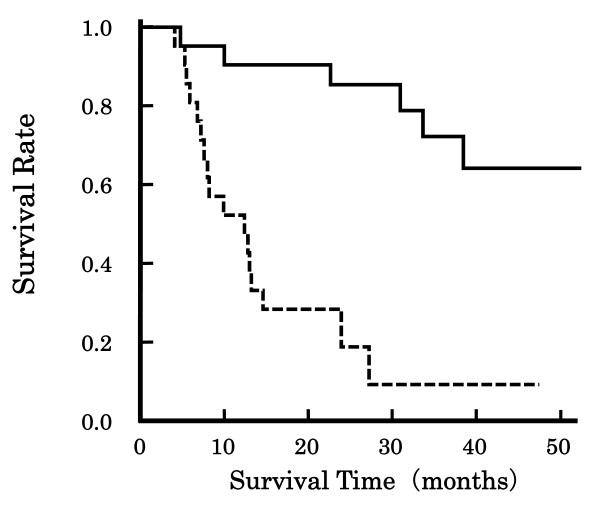
**Association of clinical response with overall survival Japanese patients with esophageal squamous cell carcinoma**. Line: CR, Dotted line: non-CR. The patients with CR survived extensively longer than the non-CR patients (p < 0.001, Log-rank test).

Table 2 indicates the association of the *TNFRSF1B *genetic polymorphisms M196R/T587G, A1466G and C1493T with clinical response in the ESCC patients. *TNFRSF1B *A1466G genotype was predictive of clinical response (p = 0.040), whereas M196R/T587G and C1493T were not. No effects of the *TNFRSF1B *genotypes were found for TNM classes and disease stage (data not shown). Figure 2 shows the association of clinical response with overall survival of the patients. The patients with CR survived markedly longer than the non-CR patients (p < 0.001, Log-rank test). However, the 2-year survival rate was 25.0%, 60.0% and 50.0% in the patients with the *TNFRSF1B *genotypes AA^1466^, AG^1466 ^and GG^1466^, and the effect of *TNFRSF1B *A1466G genotype on the overall survival was not significant (Log-rank test). In addition, the effects of *TNFRSF1B *M196R/T587G, A1466G and C1493T genotypes were not found for severe acute leucopenia, stomatitis or cheilitis (data not shown).

**Table 2:** Effects of *TNFRSF1B *polymorphisms on clinical response in Japanese patients with esophageal squamous cell carcinoma.

		Complete response N = 22	Not complete response N = 24	p
M196R/T587G (rs1061622)	TT	15	21	0.354
	TG	5	2	
	GG	2	1	
	T	35	44	0.135
	G	9	4	
				
A1466G (rs1061624)	AA	2	10	0.040
	AG	15	10	
	GG	5	4	
	A	19	30	0.094
	G	25	18	
				
C1493T (rs3397)	CC	9	12	0.787
	CT	9	9	
	TT	4	3	
	C	27	33	0.515
	T	17	15	

## Discussion

The *TNFRSF1B *gene on chromosome 1 at p36 (IBD7) consists of 10 exons and encodes 415 amino acids, whereas the *TNFRSF1A *gene at 12p13 (IBD2) consists of 10 exons and encodes 455 amino acids. TNFRSF1A is an important factor inducing apoptosis via an intracellular death domain, and TNFRSF1B is thought to be involved in ligand passing, thereby regulating the association of TNF-α with TNFRSF1A. TNFRSF1A is widely expressed, whereas TNFRSF1B is predominantly expressed in cells of the hematopoietic lineage. Several clinical investigations have been conducted to assess the predictive value of the genetic polymorphisms *TNF-α *G-308A, *TNFRSF1A *A36G and G-609T, and *TNFRSF1B *M196R/T587G, A1466G (or A1663G) and C1493T (or C1690T) regarding susceptibility to various inflammatory disorders [10-19], and recently, to cancer [23-28]. As for *TNFRSF1B*, the SNP M196R/T587G has proved predictive of Crohn's disease [13], systemic lupus erythematosus [15-17] and rheumatoid arthritis [18]. *TNFRSF1B *A1466G is not associated with Crohn's disease [13], but the haplotype 1466A-1493T might be important [11]. Recently, *TNFRSF1B *C1493T has been found to be a risk factor of tobacco-related oral carcinoma [28].

In this study, it was demonstrated that the *TNFRSF1B *A1466G genotype was a predictive factor of clinical response to treatment with a definitive 5-FU/CDDP-based chemoradiotherapy in Japanese ESCC patients. The *TNFRSF1B *G-allele at position 1466 is predictive of clinical response, whereas no such association was found for M196R/T587G or C1493T (Table 2). Clinical response was evaluated 1 month after the completion of the chemoradiotherapy, and a CR was defined as the complete disappearance of all measurable and assessable disease. Clinical response was determined by T class (an index of tumor size, p = 0.002), N class (lymph node metastasis, p = 0.007), M class (distant metastasis, p = 0.001) and disease stage (p < 0.001), but *TNFRSF1B *A1466G genotype was independent of these factors.

Clinical response was significantly associated with overall survival (Figure 2), however, *TNFRSF1B *A1466G genotype had no effect on the overall survival, presumably because it was not associated with death within 1 year after the completion of chemoradiotherapy. There is no report on the function of this polymorphism but it has been reported that higher expression levels of *TNFRSF1B *gene in colorectal cancer specimens from responding patients were observed compared with those from non-responding patients [30]. Thus, the polymorphism-dependent clinical response might be due to the polymorphism-dependent expression levels, although further studies are needed.

## Conclusions

Genetic polymorphisms of the *TNFRSF1B *gene, M196R/T587G, A1466G and C1493T, were evaluated in Japanese ESCC patients treated with a definitive 5-FU/CDDP-based chemoradiotherapy. It was found that A1466G, but not M196R/T587G or C1493T, was a predictive factor of clinical response to chemoradiotherapy. Clinical response was predicted by TNM classes and disease stage, but A1466G genotype was independent of these factors. Further clinical investigation with a large number of patients or experiments in vitro should be performed to assess the predictive value of *TNFRSF1B *A1466G genotype after chemoradiotherapy.

## Competing interests

The authors declare that they have no competing interests.

## Authors' contributions

AK, TT and TS made conception, designed and coordinated the study. MY carried out genotyping study and statistical analysis. MF and NO carried out genotyping study. TO and TT collected samples and evaluated clinical responses. AK, KK, NO, TN and TS prepared the manuscript. All authors read and approved the final manuscript.
